# A Rare Case of an Inflammatory Myofibroblastic Tumor in a Middle-Aged Female

**DOI:** 10.1155/2012/148053

**Published:** 2012-10-04

**Authors:** Lamprini Kosma, Lubna Khaldi, Panagiota Galani, Dimitrios Mytas, Sofia Lafoyianni

**Affiliations:** ^1^Department of Radiology, “Amalia Fleming” General Hospital, Athens, Greece; ^2^Department of Pathology, “Amalia Fleming” General Hospital, Athens, Greece; ^3^Department of Cardiology, Sismanoglio General Hospital, Athens, Greece

## Abstract

Inflammatory myofibroblastic tumors (IMTs) are distinct entities with specific characteristics immunohistochemically and molecularly. They are regarded as “intermediate malignancy” tumors of unknown etiology. We report a case of a 64-years-old woman with a fever and abdominal discomfort for 3 months; a computer tomography was performed indicating gastrointestinal stromal tumor. Histologically the lesion proved to be IMT of the abdomen.

## 1. Introduction

Inflammatory myofibroblastic tumors (IMTs) are relatively uncommon neoplasms. They mainly involve the lungs of children, without sex predilection, whereas, extra-pulmonary locations are manifested mostly in the viscera with a slight predominance in females [1]. Their etiology remains unknown, whereas their true origin has been widely debated regarding its neoplastic or postinflammatory nature. In the past, confusion has been presented in the literature between those tumors and wide spectrum of lesions under the term “inflammatory pseudotumors” because of their overlapping morphology. Usually IMTs are composed of myofibroblastic cells admixed with inflammatory cells [2]. The differential diagnosis includes a variety of neoplastic and reactive lesions. At present, surgery is the principal treatment [3].

Herein we report the case of a middle-aged Caucasian female with a soft tissue mass in the abdomen. A combination of clinical history, biochemical findings, and imaging features raised the suspicion of gastrointestinal stromal tumor (GIST) as initial diagnosis. However the histopathological appearance and the immunohistochemical analysis elucidated any ambiguity concerning the diagnosis of IMT. Complete surgical excision was considered sufficient and followup imperative.

## 2. Case Report

**Figure 1:**
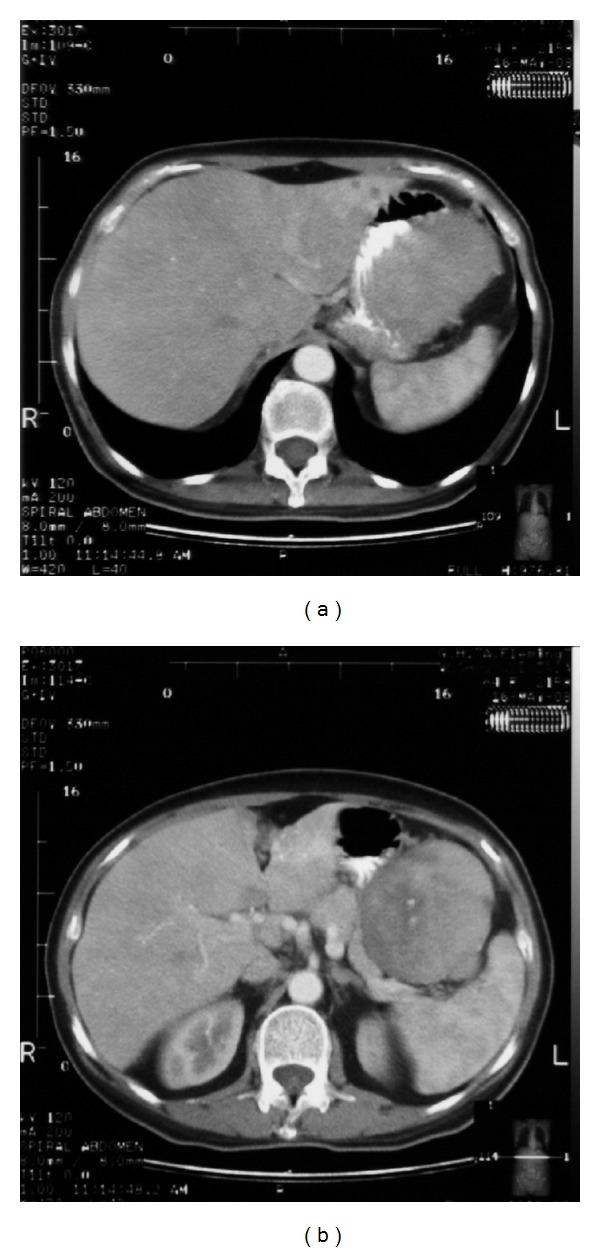
A contrast enhanced abdominal CT scan shows a large, 13.5 × 7.7 × 8.5 cm, heterogeneously enhanced mass. The mass appeared to adhere to the stomach (a). There is a clear plane between the mass and the adjacent spleen and pancreas (b).

**Figure 2:**
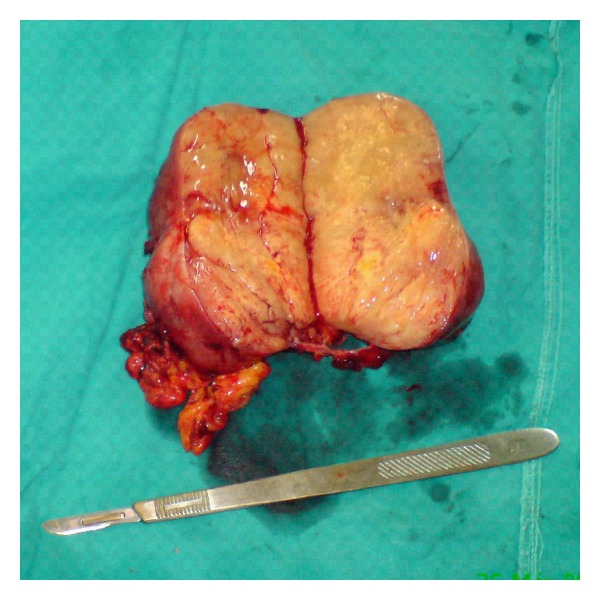
Gross appearance of the resected tumor, greatest diameter 13.5 cm.

A 64-year-old woman was referred to our hospital due to worsening fatigue, low-grade fever, and mild upper abdominal discomfort persisting for the last three months. She also reported a weight loss of about 5 kg over the last six months. During patient's hospitalization, afternoon episodes of fever with concomitant diaphoresis were registered. On physical examination a palpable mass was revealed in the left upper quadrant of the abdomen. Blood analysis showed a severe hypo-micro-anemia (HCT: 18.2%, HGB: 5.4 g/dL, MCV: 53.4 Fl, MCH: 15.8 pg, and MCHC: 29.6 g/dL) and thrombocytosis (PLT: 705 × 10^3^/*μ*L). The white blood cell (WBC) was normal. On admission laboratory tests revealed highly distorted inflammatory markers: C-reactive protein (CRP) value was 170 mg/dL and erythrocyte sedimentation rate (ESR) was 120 mm/h. Thereafter the abdominal ultrasound that was performed raised the suspicion of a mass adjusted of the spleen. An abdominal CT scan revealed a large (13.5 × 7.7 × 8.5 cm), heterogeneously enhanced mass with well defined margins. There was a clear plane between the mass and the adjacent spleen. The mass appeared to adhere to the stomach and seemed to compress the pancreas. Thus, the radiologist's initial differential diagnosis was gastrointestinal stromal cell tumor (GIST) or sarcoma (Figure 1). A complete surgical excision of the lesion was performed revealing a solid abdominal tumour clearly separated, but in close proximity to adjacent organs. The tumor was located in the left upper quadrant of the abdomen in adhesion to the major arch of the stomach and in a close proximity to the spleen, being under of the transverse colon and upwards of omentum. The blood perfusion of the tumor was supplied by neoplastic vessels originated from the contiguous organs, mainly from the spleen. None of the aforementioned organs was infiltrated by the mass. A splenectomy was performed in order to interrupt the tumour's blood supply (Figure 2).

**Figure 3:**
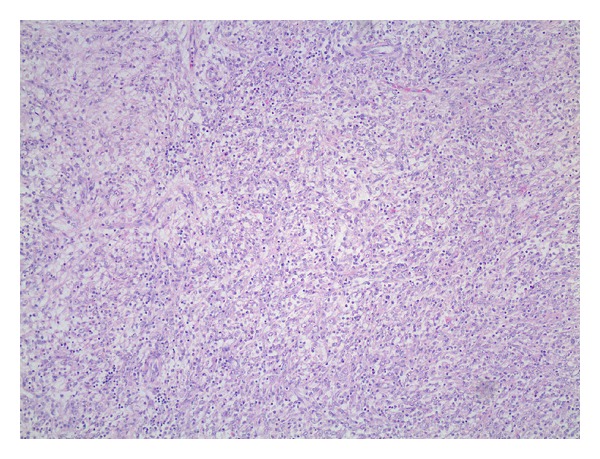
Histologic image of IMT showing chronic inflammatory cells (lymphocytes, plasma cells, and histiocytes) as well as spindle-shaped cells with pale eosinophilic cytoplasm and plump; no atypia were noticed (H/E magnification ×10).

Grossly, the mass was well demarcated measuring 13.5 cm at the greatest diameter. The capsular surface was smooth. On cut section it was lobular, with hard rubbery texture and grayish-yellow appearance. Histologically, the tumour was composed of admixture of prominent chronic inflammatory cells including lymphocytes, plasma cells and histiocytes, and spindle-shaped cells with pale eosinophilic cytoplasm and plump, focally atypical nuclei, some of which were irregular with prominent nucleoli. No significant pleomorphism was noticed (Figure 3). Immunohistochemically, it was focally positive for smooth muscle actin (SMA) and multifocally positive for desmin. Caldesmon, keratin, and ALK-1 were negative. The cytoarchitectural features along with immunophenotypical characteristics of the lesion support the diagnosis of inflammatory myofibroblastic tumour.

Postoperatively, the clinical signs and symptoms regressed, while laboratory tests gradually normalized. One month later an abdominal CT was performed showing absence of residual tissue of the preexistent tumour. Thus, no further treatment was considered necessary. After twelve months of close followup, the patient remains asymptomatic with negative laboratory and imaging tests.

## 3. Discussion

Inflammatory myofibroblastic tumors are at present rare, distinctive lesions of unknown etiology. In the past, they was referred under various synonymous such as plasma cell granuloma, xanthomatous pseudotumor, pseudosarcomatous myofibroblastic proliferation, myofibroblastoma, inflammatory myofibrohistiocytic proliferation, and most commonly inflammatory pseudotumor [4]. Hence, a scientific confusion existed for several years and accurate data were difficult to be obtained. In recent literature IMTs have emerged as a distinct entity, being classified by World Health Organization as tumors of intermediate biological potential due to a tendency for local recurrence and its small risk for distant metastasis [5]. 

Histologically composed of cellular, fascicular fibroblastic/myofibroblastic proliferations accompanied by prominent infiltrate of chronic inflammatory cells particularly plasma cells. The spindle cell component has plump focally atypical nuclei and variable mitotic rate [1]. It has been suggested that histologic criteria for malignant transformation included increased cellularity, increased numbers of ganglion-like plump polygonal or round cells associated with necrosis, large prominent nucleoli, and variable numbers of mitoses, including atypical mitotic figures and overexpression of p53 [5–7]. Ultrastructural studies indicated that the main feature of these tumors is the myofibroblast justifying the term “*inflammatory myofibroblastic tumors*.” Therefore, on frozen sections, it is unsafe to differentiate this tumor from an inflammatory process.

Contradiction appeared in the literature concerning features that will predict the biological behavior of this entity. Biselli et al. in their studies investigated the DNA ploidy status of those tumors and concluded that aneuploid IMTs possibly have malignant behavior, also DNA flow cytometry is a reliable tool that allows better diagnostic and prognostic evaluation of IMTs [6]. Others described the histologic evolution to a higher grade as malignant transformation. Those parameters are no longer valid; in fact the outcome unfortunately is not reliably predictable on morphological grounds [3, 7]. 

The histogenesis of IMTs is uncertain; some researchers supposed that IMTs are reactive lesions and implicated several factors such as surgery, trauma, ventriculo-peritoneal shunts, radiotherapy, steroids, and infectious agents, without convincing arguments [8]. Others suggested that IMTs are probably true neoplasms rather than postinflammatory processes because of cytogenetic clonality, recurrent involvement of chromosomal region 2p23, occasional aggressive local behavior, and metastasis of the tumor [6, 9]. Coffin et al. in their previous study demonstrate that subset of IMTs are neoplasms with consistent clonal aberrations involving the short arm of chromosome 2 in the region p21–p23 whereas a second subset lacks evidence of such abnormalities but is aneuploid and potentially more aggressive [10]. Additionally, there are no distinguishing histologic characteristics to explain the heterogeneity or variations in clinical features [10, 11]. 

Clinically, IMTs are presented with common symptoms such as fever, night sweating, malaise, weight loss is and anemia. Laboratory findings are nonspecific; abnormality often observed in elevation of CRP and ESR and/or increase of WBC count, reflecting the inflammatory characteristics of the tumor [12]. The clinical and laboratories findings of our patient were analogous, but the diagnosis of IMT on the basis of those findings was precarious. Nevertheless, the question remained whether those findings can be caused by the lesion. Thus the patient was referred to radiology department to determine the nature of the palpable abdominal mass. 

On CT scan IMT tumors frequently presented as a circumscribed soft tissue mass, with strong heterogeneous enhancement. The latter is variable, depending on the balance between the cellular component and the fibrous tissue. Calcification, hemorrhage, necrosis, and aggressive features such as invasion of adjacent tissues or bones' erosion may be found in a minority of cases. Tumors dimension range from 1 cm to greater than 20 cm, with a mean size of 6 cm [13]. However, those findings are not specific and are applied to other entities as well. The CT scan of our case was similar to those described in the literature, explaining probably the cause of fever, weight loss, and anemia, but the nature of the lesion remained undiagnosed due to imaging nonspecificity. An abdominal ultrasound was also performed, which, in our patient, did not clarify the ambiguity. Thus, we confirmed that IMTs do not have any specific ultrasound findings.

Another diagnostic dilemma to be faced was the location of the mass. Most common anatomical location of IMTs is the lung. The mass of our patient was adhered to the stomach and caused a displacement of the pancreas and the spleen. Thus, its contiguity with the adjacent stomach made the differential diagnosis limited. What was the nature of the mass? Was the tumor displacing the stomach or was it originating from the stomach? Our differential diagnoses on imaging bases included mainly a gastrointestinal stromal cell tumor (GIST) and a sarcoma.

According to English literature, IMTs have a predilection for children and adolescents. In some rare cases it may involve any anatomical part and may arise as late as the eighth decade of life [5, 14, 15]. However, Gleason and Hornick emphasized that the diagnosis of IMT in middle aged or elderly patients should be made with hesitance [5]. In our case a slight contradiction to literature was evident, for she was middle aged and the mass was located in the abdomen. Hence, the age of the patient, the location of the mass, the vagueness of clinical presentation, and the nonspecificity of radiological findings contributed to exclude IMT from the initial diagnosis. 

Surgical excision with clear resection margins is proposed as the principle treatment in all cases [11]. Local recurrences were associated with abdominopelvic site, larger size, and older age, occuring in about 10% to 25% cases of abdominal tumors especially within a year of surgery. Distant metastases were associated with younger age, larger size, and both abdominopelvic and pulmonary sites. Estimated as less as 5% with predilection for lung, brain, liver, and bone, are appeared at presentation up to 9 years later [1, 3, 5]. Chemotherapy, radiotherapy, and corticosteroid administration have been suggested without efficient results or any beneficiary of those modalities [8]. Prognostic factors are uncertain, at present; absence of ALK reactivity is associated with the development of metastases [3]. Our patient had a complete surgical excision of the tumour which was considered sufficient. Although the tumour was negative for ALK, a 12-month followup did not indicate metastatic disease or recurrence. 

In conclusion, IMTs are uncommon, true neoplasms with biological behavior that range in most cases from benignancy to the rare aggressive variants. Final diagnosis should be based on histomorphological features and immunohistochemical analysis. Morphology is not a reliable parameter to predict the outcome. Complete surgical excision and a long-term multidisciplinary followup is the most indicated therapeutic approach. Appropriate awareness should be exercised by radiologists to abdominal solid tumors in combination with constitutional symptoms and abnormal hematologic and serologic findings, to avoid misdiagnoses.
